# An Effective Big Data Supervised Imbalanced Classification Approach for Ortholog Detection in Related Yeast Species

**DOI:** 10.1155/2015/748681

**Published:** 2015-10-29

**Authors:** Deborah Galpert, Sara del Río, Francisco Herrera, Evys Ancede-Gallardo, Agostinho Antunes, Guillermin Agüero-Chapin

**Affiliations:** ^1^Departamento de Ciencias de la Computación, Universidad Central “Marta Abreu” de Las Villas (UCLV), 54830 Santa Clara, Cuba; ^2^Department of Computer Science and Artificial Intelligence, Research Center on Information and Communications Technology (CITIC-UGR), University of Granada, 18071 Granada, Spain; ^3^Centro de Bioactivos Químicos, Universidad Central “Marta Abreu” de Las Villas (UCLV), 54830 Santa Clara, Cuba; ^4^Centro Interdisciplinar de Investigação Marinha e Ambiental (CIMAR/CIIMAR), Universidade do Porto, Rua dos Bragas 177, 4050-123 Porto, Portugal; ^5^Departamento de Biologia, Faculdade de Ciências, Universidade do Porto, Rua do Campo Alegre, 4169-007 Porto, Portugal

## Abstract

Orthology detection requires more effective scaling algorithms. In this paper, a set of gene pair features based on similarity measures (alignment scores, sequence length, gene membership to conserved regions, and physicochemical profiles) are combined in a supervised pairwise ortholog detection approach to improve effectiveness considering low ortholog ratios in relation to the possible pairwise comparison between two genomes. In this scenario, big data supervised classifiers managing imbalance between ortholog and nonortholog pair classes allow for an effective scaling solution built from two genomes and extended to other genome pairs. The supervised approach was compared with RBH, RSD, and OMA algorithms by using the following yeast genome pairs: *Saccharomyces cerevisiae*-*Kluyveromyces lactis*, *Saccharomyces cerevisiae*-*Candida glabrata*, and *Saccharomyces cerevisiae*-*Schizosaccharomyces pombe* as benchmark datasets. Because of the large amount of imbalanced data, the building and testing of the supervised model were only possible by using big data supervised classifiers managing imbalance. Evaluation metrics taking low ortholog ratios into account were applied. From the effectiveness perspective, MapReduce Random Oversampling combined with Spark SVM outperformed RBH, RSD, and OMA, probably because of the consideration of gene pair features beyond alignment similarities combined with the advances in big data supervised classification.

## 1. Introduction 

Orthologs are defined as genes in different species that descend by speciation from the same gene in the last common ancestor [1]. Their probable functional equivalence has made them important for genome annotation, phylogenies, and comparative genomics analyses. Ortholog detection (OD) algorithms should distinguish orthologous genes from other types of homologs such as paralogs evolving from a common ancestor through a duplication event. A great deal of unsupervised graph-based [2–8], tree-based [9–13], and hybrid approaches [14, 15] have been developed to identify orthologs resulting in corresponding repositories for precomputed orthology relationships.

Focusing on the graph-based approach, orthogroups are generally built from the comparison of genome pairs by using BLAST searches [16] and then the application of some “nearest neighbor” heuristics such as Best BLAST Hit (Bet) [2], Bidirectional Best Hit (BBH) [17], Reciprocal Best Hits (RBH) [18], Reciprocal Smallest Distance (RSD) [19], or Best Unambiguous Subset (BUS) [20] to find potential pairwise orthology relationships. Subsequently, algorithms can return pairwise relationships, if they perform pairwise ortholog detection (POD) such as RBH [18] and RSD themselves [19], and Comprehensive, Automated Project for the Identification of Orthologs from Complete Genome Data (OMA) Pairwise [21], or they can apply clustering to predict orthogroups from the score of the alignment process.

When OD is based only on sequence similarity, it has been limited by evolutionary processes such as recent paralogy events, horizontal gene transfers, gene fusions and fissions, domain recombinations, or different genetic events [22, 23]. In fact, the identification of homologs is a difficult task in the presence of short sequences, those that evolved in a convergent way and the ones that share less than 30% of amino acid identities (twilight zone). Algorithm failures have been particularly shown in benchmark datasets from* Saccharomycetes* yeast species that underwent whole genome duplications (WGD) and, certainly, present rampant paralogy and differential gene losses [24].

To tackle these shortcomings for OD, some OD solutions may integrate the conserved neighborhood (synteny) of genes in the inference process for related species. Currently, there is a tendency of merging sequence similarity with synteny [20, 25, 26] genome rearrangements [27, 28], protein interactions [15], domain architectures [29], and evolutionary distances [19]. However, so far there is no report that combines such features in a supervised approach to increase POD effectiveness.

On the other hand, the integration of different gene or protein information and the massive increase in complete proteomes highly increase the dimensionality of the OD problem and the total number of proteins to be classified. In a thorough paper from the Quest for Orthologs consortium [30], the authors emphasize the idea that this increase in proteome data brings out the need to work out not only efficient but effective OD algorithms. As they mention, the increase in computational demands in sequence analyses is not easily met by an increase in computational capacities but rather calls for new approaches or algorithmic implementations [30]. In this sense, they summarized some methodological shortcuts implemented by the existing orthology databases to deal with the scaling problem.

Considering all these previous remarks about OD, we propose a new supervised approach for pairwise OD (POD) that combines several gene pairwise features (alignment-based and synteny measures with others derived from the pairwise comparison of the physicochemical properties of amino acids) to address big data problems [30]. Our big data supervised POD approach allows scaling to related species and data imbalance management (low ortholog ratio found in two or more genomes) for an effective OD. The methodology consists of three steps:

- The calculation of gene pair features to be combined.
- The building of the classification model using machine learning algorithms to deal with big data from a pairwise dataset.
- The classification of related gene pairs.

Since traditional supervised classifiers cannot scale large datasets, the supervised classification for the POD problem should be addressed as a big data classification problem according to [31–33] and big data solutions should be applied for binary classification in imbalanced data such as the ones presented in [34] based on MapReduce [35].

Finally, we evaluate the application of several big data supervised techniques that manage imbalanced datasets [34, 36] such as cost-sensitive Random Forest (RF-BDCS), Random Oversampling with Random Forest (ROS + RF-BD), and the Apache Spark Support Vector Machines (SVM-BD) [36] combined with MapReduce ROS (ROS + SVM-BD). The effectiveness of the supervised approach is compared to the well-known unsupervised RBH, RSD, and OMA algorithms following an evaluation scheme that takes data imbalance into account. All the algorithms were evaluated on benchmark datasets derived from the following yeast genome pairs:* S. cerevisiae* and* K. lactis*,* S. cerevisiae* and* C. glabrata* [24], and* S. cerevisiae* and* S. pombe* [37]. The* S. cerevisiae* and* C. glabrata *pair is particularly complex for OD since both species had undergone WGD. We found that our supervised approach outperformed traditional methods, mainly when we applied ROS combined with SVM-BD.

## 2. Materials and Methods 

### 2.1. Gene Pair Features

Starting from two genome representations being *G*_1_ = {*x*_1_, *x*_2_,…, *x*_*n*_} and *G*_2_ = {*y*_1_, *y*_2_,…, *y*_*m*_}, with *n* and *m* annotated gene sequences or proteins, respectively, we define gene pair features in Table 1 representing continuous normalized values of the following similarity measures:

(i) The sequence alignment measure *S*_1_ averages the local and global protein alignment scores from the Smith Waterman [38] and the Needleman-Wunsch [39] algorithms calculated with a specified scoring matrix and “gap open” (GOP) and “gap extended” (GEP) parameters.
(ii) Measure *S*_2_ is calculated from the length (*L*) of the sequences by using the normalized difference for continuous values [40].
(iii) The similarity measure *S*_3_ is calculated from the distance between pairs of sequences in regard to their membership to locally collinear blocks (LCBs). These blocks represent truly homologous regions that can be obtained with the Mauve software [41]. The LCB[*k*, 1 ⋯ *n*] matrix represents the total number of codons in the block *k* for each *n* gene belonging to genome *G*_1_; and LCB[*k*, *n* ⋯ *n* + *m*] counts for the membership in genome *G*_2_. The total number of LCBs where one or both of the sequences in the gene pair (*x*_*i*_, *y*_*j*_) contain at least one codon is represented by *Q*. The normalized difference is selected for the comparison of the continuous values in the LCB[*k*, *p*] matrix.
(iv) Based on the spectral representation of sequences from the global protein pairwise alignment, the *S*_4_ measure uses the Linear Predictive Coding [40]. First, each amino acid that lies in a matching region without “gaps” between two aligned sequences is replaced by its contact energy [42]. The average of this physicochemical feature in the predefined window size *W*, called the moving average for each spectrum, is then calculated. Next, the similarity measure Corr(*MX*, *MY*) between the two spectral representations in a matching region is calculated by using the Pearson correlation coefficient and the corresponding significance level. Finally, the significant similarities of the *R* regions without “gaps” are aggregated considering the length len_*k*_ of each *k* region. From our previous studies presented in [43, 44], we have considered three features for the physicochemical profile with *W* values of 3, 5, and 7.

### 2.2. Big Data Supervised Classification Managing Data Imbalance

Given a set *A* = {*S*_*r*_(*x*_*i*_, *y*_*j*_)} of gene pair features or attributes as discrete or continuous values of *r* gene pair similarity measure functions, previously specified, we represent a POD decision system DS = (*U*, *A* ∪ {*d*}), where *U* = {(*x*_*i*_, *y*_*j*_)}, ∀*x*_*i*_ ∈ *G*_1_ and ∀*y*_*j*_ ∈ *G*_2_, is the universe of the gene pairs and *d* ∉ *A* is the binary decision attribute obtained from a curated classification. This decision attribute defines the extreme data imbalance. Given an underlying function *f* : *S* → {0,1} defined on the set *S* of gene pair instances, the learning process produces a set of learning functions $\Gamma =\{\hat{f}:L\to \left\{0,1\right\}|L\subset S\}$ that approximate *f* from the train set *L*. The goal is to find the best approximation function from Γ having a fitness function or a classification evaluation metric. In this case, the evaluation metric should take into account the low ratio of orthologs to the total number of possible gene pairs in the test set (*S*-*L*). The big data supervised classification divides *S* into train and test instance to build a learning model $\hat{f}$ and to classify the instances by means of a big data supervised algorithm managing the imbalance between classes.

The proposed big data processing framework is shown in Table 2. We use the open-source project Hadoop [45] with its highly scalable and fault-tolerant Hadoop Distributed File System (HDFS). We also utilize the scalable Mahout data mining and machine learning library [46] with machine learning algorithms adapted according to the MapReduce scheme as the MapReduce implementation of the RF algorithm [47]. Finally, we use the Apache Spark framework [36] interacting with HDFS, when the implementation of SVM-BD in the scalable MLLib machine learning library [48] is combined with the MapReduce ROS implementation [34].

### 2.3. Evaluation Scheme Considering Data Imbalance

**Figure 1:**
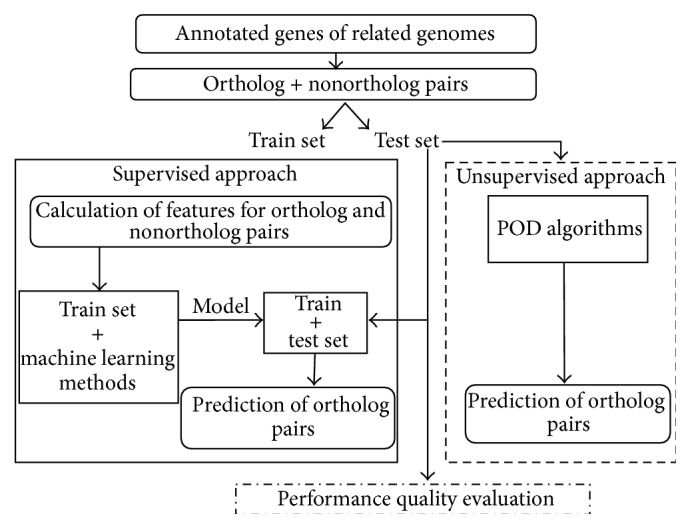
Workflow of the evaluation of supervised versus unsupervised POD algorithms.

For the evaluation of POD algorithms, we compare the supervised solutions and the unsupervised ones represented by the reference RBH, RSD, and OMA algorithms following the evaluation scheme in Figure 1. The process separates the pairs into train and test sets and calculates pairwise similarity measures for the pairs of both sets. The sequences of the test sets should be used to run the unsupervised reference algorithms. The train set should be used for building the supervised models to be tested only with the test set.

The performance quality evaluation involves the calculation of the following evaluation metrics for imbalanced datasets.

The geometric mean (*G-*Mean) [49] is defined as

(1) $$\begin{array}{c} G\text{-Mean}=\sqrt{\text{sensitivity}*\text{specificity}}, \end{array}$$

where sensitivity = TP/(TP + FN) and TN_Rate_ = specificity = TN/(FP + TN) are calculated from true positives (TP), false negatives (FN), false positives (FP), and true negatives (TN).

The Area Under the ROC Curve (AUC) [50] is computed obtaining the area of the ROC graphic. Concretely, we approximate this area using the average of true positive rate and false positive rate values by means of the following equation:

(2) $$\begin{array}{c} \text{AUC}=\frac{1+{\text{TP}}_{\text{rate}}-{\text{FP}}_{\text{rate}}}{2}, \end{array}$$

where TP_rate_ = TP/(TP + FN) corresponds to the percentage of positive instances correctly classified and FP_rate_ = FP/(FP + TN) corresponds to the percentage of negative instances misclassified.

We use *G-*Mean seeking to maximize the accuracy of the two classes (orthologs and nonorthologs) by achieving a good balance between sensitivity and specificity that consider misclassification costs and AUC to show the classifier performance over a range of data distributions [51].

### 2.4. Experiments for Building and Testing the Supervised POD Algorithms

#### 2.4.1. Datasets

For the evaluation of POD algorithms in related yeast genomes, in Experiment 1 we evaluated the algorithms inside a genome by partitioning at random 75% of the complete set of pairs for training and 25% for testing, and in Experiment 2 we built the model from a genome pair and tested it in two different pairs. Specifically, in Experiment 1 we divided the* S. cerevisiae*-*K. lactis *set into 16.986.996 pairs for training and 5.662.332 pairs for testing. The four datasets (Blosum50, Blosum621, Blosum622, and Pam250) of each genome pair, summarized in Tables 3, 4, and 5, were built from combinations of alignment parameter settings shown in Table 6. On the other hand, in Experiment 2, we built the classification model from 22.649.328 pairs of* S. cerevisiae* and* K. lactis* genomes and tested it in 29.887.416 pairs of* S. cerevisiae *and* C. glabrata* and 8.095.907 pairs of* S. cerevisiae *and* S. pombe *genomes.

*S. cerevisiae*-*S. pombe* dataset contains ortholog pairs representing 95.18% of the union of the Inparanoid7.0 and GeneDB classifications described in [37]. On the other hand,* S. cerevisiae*-*K. lactis* and* S. cerevisiae*-*C. glabrata *datasets contain all ortholog pairs in the gold groups reported in [24]. When we built the set of instances with all possible pairs, we just excluded 89 genes from* S. cerevisiae*, 37 from* C. glabrata*,and 1403 from* K. lactis* since we did not find their genome physical location data in the YGOB database [52], required for the LCB feature calculation.

Tables 3, 4, and 5 summarize the characteristics of the four datasets including the total number of gene pairs (#Ex.), the number of attributes (#Atts.), the labels for majority and minority classes (Class (maj; min)), the number of pairs in both classes (#Class (maj; min)), the percentage of pairs in majority and minority classes (%Class (maj; min)), and the imbalance ratio (IR).

The calculation of gene pair features or attributes (average of local and global alignment similarity measures, length of sequences, gene membership to conserved regions (synteny), and physicochemical profiles within 3, 5, and 7 window sizes) was specified in the previous section.

#### 2.4.2. Algorithms and Parameter Values

The supervised algorithms compared in the experiments and the parameter values are specified in Table 7. Additionally, Table 8 summarizes the parameter values and the implementation details for the unsupervised algorithms.

## 3. Results and Discussion

In this section, we first analyze the supervised approaches based on big data technologies, and later we compare the best supervised solution with the classical unsupervised methods.

### 3.1. Supervised Classifiers: Analysis of Big Data Based Approaches

The *G-*Mean values of the supervised classifiers with the best performance in Experiments 1 and 2 are shown in Table 9 for the Blosum50, Blosum621, Blosum622, and Pam250 datasets. The best values are in boldface. The *G-*Mean values of the supervised algorithms change only slightly with the selection of different alignment parameters. The stability of these classification results may be caused either by the aggregation of global and local alignment scores in a single similarity measure or by the appropriate combination of scoring matrices and gap penalties in relation to the sequence diversity between the two yeast genomes. The selection of the four scoring matrices was aimed at finding homologous protein sequences in a wide range of amino acid identities between both genomes. For example, Blosum50 and Pam250 scoring matrices are frequently used to detect proteins sharing less than 50% of amino acid identities [53]. In addition, the selected gap penalties values are not low enough to affect the sensitivity of the alignment [53].

**Figure 2:**
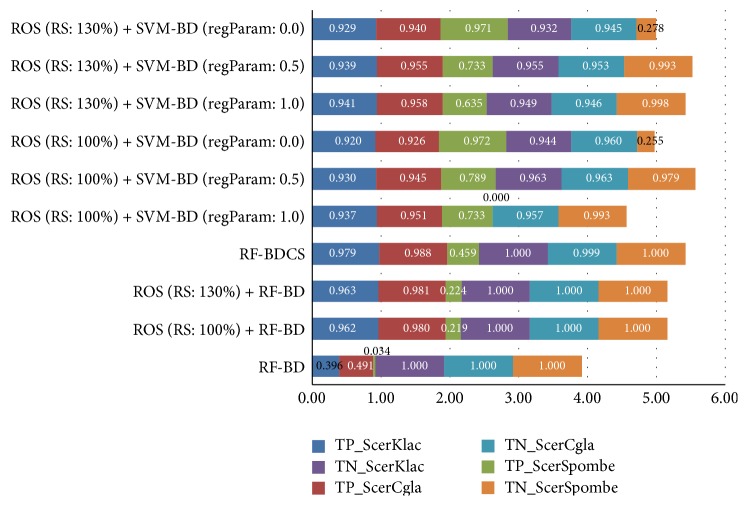
Average true positive and true negative rate values of supervised classifiers obtained in Experiments 1 and 2.

The average results of AUC and *G-*Mean obtained in Experiments 1 and 2 for the supervised algorithms with different parameter values are shown in Table 10. The average TP_Rate_ and TN_Rate_ are also depicted in Figure 2. SVM-BD has been left out from the table due to its very poor performance in *G-*Mean caused by its imbalance between TP_Rate_ and TN_Rate_ as shown in Figure 2. Both Table 10 and Figure 2 prove that big data supervised classifiers managing imbalance outdo their corresponding big data supervised versions.

The ROS preprocessing method for big data makes SVM-BD useful for POD and improves the performance of RF-BD even more with a higher value for the resampling size parameter of 130% [54]. In contrast, both experiments show that the variation in this parameter value from 100% to 130% does not significantly influence the performance of the SVM-BD classifier with different regulation values.

Specifically, RF-BDCS shows the best performance in* S. cerevisiae*-*C. glabrata* and* S. cerevisiae*-*K. lactis* when the classification quality is measured by *G-*Mean and AUC metrics, because it enhances the learning of the minority class. The criterion used to select the best tree split is based on the weighting of the instances according to their misclassification costs, and such costs are also considered to calculate the class associated with a leaf [34]. This cost treatment does not explicitly change the sample distribution and avoids the possible overtraining that it is present in the ROS solutions due to replicated cases. The election of the cost values (*C*(+∣−) = IR and *C*(−∣+) = 1) may also define the success of the algorithm.

In the case of SVM-BD, the fixed regularization parameter defines the trade-off between the goal of minimizing the training error (i.e., the loss) and minimizing the model complexity to avoid overfitting. The higher its value, the simpler the model. Nonetheless, setting an intermediate value or one close to zero may produce a better performance in classification [48]. This is the case of the ROS (RS: 100%) + SVM-BD (regParam: 0.5) classifier that exhibits the best AUC and *G-*Mean values in* S. cerevisiae*-*S. pombe* and the best balance between TP_Rate_ and TN_Rate_ in the three datasets (Figure 2).

In order to balance time with classification quality, time consumption is another aspect to have in mind when comparing big data solutions.  Table 11 contains run time in seconds for all big data solutions in each dataset and the faster algorithms are highlighted in boldface. These results allow us to prove that the time required is directly related to the operations needed for each method, as well as to the size of the datasets used to build the model. The fastest algorithm considering the average run time is SVM-BD followed by SVM-BD combined with ROS. Thus, the fastest algorithms coincide with the ones with better performance. In general, the ROS (RS: 100%) + SVM-BD (regParam: 0.5) classifier can be considered the best supervised solution considering both performance and time.

### 3.2. Comparison of Supervised* versus* Unsupervised Classifiers

**Figure 3:**
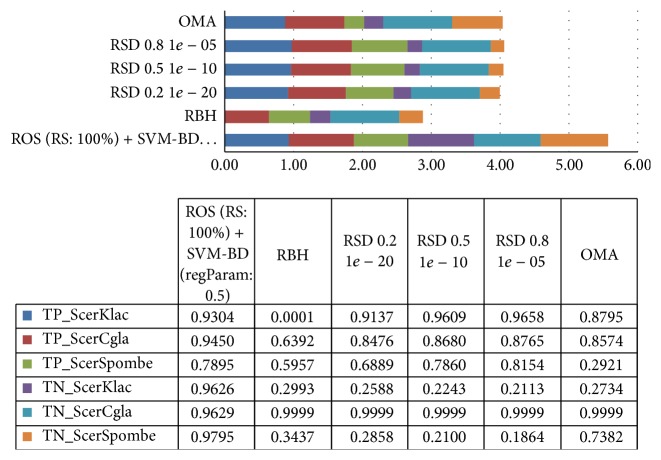
Average true positive and true negative rate values of the unsupervised and the best supervised classifiers in Experiments 1 and 2.

The average results of AUC and *G-*Mean obtained for the best supervised algorithms and the unsupervised algorithms with different parameter values are shown in Table 12 for Experiments 1 and 2. The average TP_Rate_ and TN_Rate_ are also depicted in Figure 3. The supervised classifiers outperform the unsupervised ones. Among the unsupervised algorithms, RSD reaches the highest* G-*Measure value by setting *E*-value = 1*e* − 05 and *α* = 0.8 (recommended values in [55]) in* S. cerevisiae*-*C. glabrata* where similar results can also be seen for AUC and TP_Rate_ values. On the contrary, OMA was the best among the unsupervised algorithms in* S. cerevisiae*-*S. pombe *datasets (Table 12).

In general, the performance of all classifiers declined in* S. cerevisiae*-*S. pombe* datasets due to the fact that* S. pombe* is a distant relative of* S. cerevisiae *[56]. The supervised classifiers performance is affected for the same reason and also by the difference in data distribution between the train and test sets [57]. Conversely, ROS (RS: 100%) + SVM-BD (regParam: 0.5) remained stable in* S. cerevisiae*-*C. glabrata* and* S. cerevisiae*-*S. pombe* datasets when considering the balance between TP_Rate_ and TN_Rate_. Superior results in* S. cerevisiae*-*C. glabrata *are outstanding, since both genomes underwent WGD and a subsequent differential loss of gene duplicates, so that algorithms are prone to produce false positives. Thus, this dataset contains “traps” for OD algorithms [24].

The reduced quality shown by RBH, RSD, and OMA, mainly in the case of RBH, could be caused by their initial assumption that the sequences of orthologous genes/proteins are more similar to each other than they are to any other genes from the compared organisms. This assumption may produce classification errors [22], mainly in RBH, that infer orthology relationships simply based on reciprocal BLAST Best Hits, in spite of the fact that BLAST parameters can be tuned as has been recommended in [58].

Conversely, RSD not only compares the sequence similarity of query sequence *a* of genome *A* against all sequences of genome *B* using the BLASTp algorithm, but also separately aligns sequence *a* against the corresponding set of hits resulting from a BLAST search. Those pairs that satisfy a divergence threshold (defined as the fraction of the alignment total length) are used for the calculation of evolutionary distances. From this step, sequence *b* yielding the shortest distance with sequence *a* is retained and then used as query for a reciprocal BLASTp against genome *A*. Thus, the algorithm is repeated in the opposite direction, and if *b* finds *a* as its best reciprocal short distance hit, then the pair (*a*, *b*) can be assumed as an ortholog pair and their evolutionary distance is retained. In sum, the RSD procedure relies on global sequence alignment and maximum likelihood estimation of evolutionary distances to detect orthologs between two genomes, and as a result, it finds many putative orthologs missed by RBH because it is less likely than RBH to be misled by existing close paralogs.

The OMA algorithm also displays advantages over RBH, corroborated in both Experiments 1 and 2. It uses evolutionary distances instead of alignment scores. This algorithm allows the inclusion of one-to-many and many-to-many orthologs. It also considers the uncertainty in distance estimations and detects potential differential gene losses.

From the point of view of the intrinsic information managed by the algorithms, the success of big data supervised classifiers managing imbalance over RSD and OMA may be explained by feature combinations calculated for the datasets together with the learning from curated classifications. That is, the assembling of alignment measures together with the comparison of sequence lengths, the membership of genes to conserved regions (synteny), and the physicochemical profiles of amino acids improves the supervised classification results on the test sets, even in those built from two species that underwent WGD.

With the aggregation of global and local alignment scores, we are combining protein structural and functional relationships between sequence pairs, respectively. Besides, we incorporate other gene pair features: (i) the periodicity of the physicochemical properties of amino acids which allows us to detect similarity among protein pairs in their spectral dimension [59]; (ii) the conserved neighborhood information, which considers that genes belonging to the same conserved segment in genomes of different species will probably be orthologs; and (iii) the length of sequences that can be seen as the relative positions of nucleotides/amino acids within the same gene/protein in different species and in duplicated genomic regions within the same species.

In order to obtain (i), each of the two aligned sequences is first represented as an ordered arrangement of moving average values of amino acids contact energies in a window frame of the aligned regions without gaps. Then, each spectrum is correlated to obtain the pair similarity value. This feature may allow us to deal with sequences having functional similarities despite their low amino acid sequence identities (<35%). These sequences may affect OD in* S. cerevisiae*-*S. pombe* which are moderately related and their orthologs may be diverged.

In feature (ii), two genes from different genomes are more likely to be orthologs when they share a high sequence similarity and they are placed in the same LCB (conserved segment that does not seem to be altered by genome rearrangements [60]). The detection of authentic orthologs is frequently impaired by genome rearrangements and other large-scale evolutionary events like WGD.

With regard to sequence length (iii), it is disturbed by insertion and deletion of stretches of DNA over evolutionary time. This makes more distant relatives have a higher likelihood of sequence length difference [61]. In this way, the genomes involved in this study are relatives and length similarities may complement the detection of homology.

## 4. Conclusions

The development of effective supervised algorithms for POD in a big data scenario was made possible by (i) the availability of curated databases (authentic orthologs), (ii) the combination of traditional alignment measures with other gene pair features (sequence length, gene membership to conserved regions, and physicochemical profiles) to complement homology detection, and (iii) the treatment of the low ratio of orthologs to the total possible gene pairs between two genomes. By applying evaluation metrics such as *G-*Mean, AUC, and the balance between TP_Rate_ and TN_Rate_, our results show that gene pairwise feature combinations provide excellent POD in a big data supervised scenario that considers data imbalance. The SVM-BD classifier combined with the ROS (RS: 100%) preprocessing with regulation parameter 0.5 outdid the rest of the big data supervised solutions and the popular unsupervised (RBH, RSD, and OMA) algorithms even when the supervised model was extended to datasets containing “traps” for OD algorithms. The classification performance of the supervised algorithms measured by *G-*Mean and AUC metrics did not significantly change in the four test sets obtained with different alignment parameter settings. When the balance between time and classification quality is considered, ROS (RS: 100%) + SVM-BD (regParam: 0.5) also proves to be the algorithm of choice.

In future research, the introduction of new gene pair features might improve the effectiveness and efficiency of the supervised algorithms for POD.

## Figures and Tables

**Table 1:** Gene pair features.

Measure	Definition	Parameters
Local and global alignment	$S_{1}\left(x_{i},y_{j}\right)=\frac{S_{l}\left(x_{i},y_{j}\right)+S_{g}\left(x_{i},y_{j}\right)}{2}$ $S_{l}\left(x_{i},y_{j}\right)=\left\{\begin{array}{cc} c_{l}\left(x_{i},y_{j}\right), & c_{l}\left(x_{i},y_{j}\right)>0\\ 0, & c_{l}\left(x_{i},y_{j}\right)\leq 0 \end{array}\right.$ $c_{l}\left(X_{i},Y_{j}\right)=\frac{\text{swalign}\left(x_{i},y_{j},M,go,ge\right)}{\max\left(\text{swalign}\left(x_{k},y_{p},M,go,ge\right)\right)}$, ∀*k* ∈ [1, *n*], ∀*p* ∈ [1, *m*] $S_{g}\left(X_{i},Y_{j}\right)=\left\{\begin{array}{cc} c_{g}\left(x_{i},y_{j}\right), & c_{g}\left(x_{i},y_{j}\right)>0\\ 0, & c_{g}\left(x_{i},y_{j}\right)\leq 0 \end{array}\right.$ $c_{g}\left(X_{i},Y_{j}\right)=\frac{\text{nwalign}\left(x_{i},y_{j},M,go,ge\right)}{\max\left(\text{nwalign}\left(x_{k},y_{p}M,go,ge\right)\right)}$, ∀*k* ∈ [1, *n*], ∀*p* ∈ [1, *m*]	*M*-substitution matrix and go, ge-GOP, and GEP

Length	$S_{2}\left(x_{i},y_{j}\right)=1-\frac{\left|L\left(x_{i}\right)-L\left(y_{j}\right)\right|}{\max\left(L\left(z_{k}\right)\right)-\min\left(L\left(z_{k}\right)\right)}$, *z* = *x*_1_, *x*_2_,…, *x*_*n*_, *y*_1_, *y*_2_,…, *y*_*m*_ ∀*k* ∈ [1, *n* + *m*]	

Membership to locally collinear blocks	*S*_3_(*x*_*i*_, *y*_*j*_) = 1 − *d*_lcb_(*x*_*i*_, *y*_*j*_) $d_{lcb}\left(x_{i},y_{j}\right)=\frac{1}{Q}\times \sum_{k=1}^{LCBs}d_{lcb}\left({k,x}_{i},y_{j}\right)$ $d_{lcb}\left({k,x}_{i},y_{j}\right)=\left\{\begin{array}{cc} 0, & \max\left(LCB\left[k,p\right]\right)=\max\left(LCB\left[k,p\right]\right)\\ \frac{\left|LCB\left[k,i\right]-LCB\left[k,n+j\right]\right|}{\max\left(LCB\left[k,p\right]\right)-\min\left(LCB\left[k,p\right]\right)}, & \forall p\in \left[1,n+m\right];\;\;k=1\cdots LCBs \end{array}\right.$	Mauve software parameters

Physicochemical profile	$\text{Corr}\left(MX,MY\right)=\left\{\begin{array}{cc} \text{Corr}\left(MX,MY\right), & \text{sig}\leq 0.05\\ 0, & \text{sig}>0.05 \end{array}\right.$ $S_{4}\left(x_{i},y_{j}\right)=\frac{\sum_{k=0}^{R}\text{Corr}\left({MX}_{ik},{MY}_{jk}\right)\times {\text{len}}_{k}}{\sum_{k=0}^{R}{\text{len}}_{k}}$	*W-*moving average window size of each spectrum

**Table 2:** Big data framework, applications, and algorithms.

Big data framework	Application	Algorithms
Hadoop 2.0.0 (Cloudera CDH4.7.1) with the head node configured as name-node and job-tracker, and the rest as data-nodes and task-trackers	(i) MapReduce ROS implementation (ii) A cost-sensitive approach for Random Forest MapReduce algorithm (RF-BD) (iii) MapReduce RF implementation (Mahout library)	RF-BDCS ROS (100%) + RF-BD ROS (130%) + RF-BD

Apache Spark 1.0.0 with the head node configured as master and name-node, and the rest as workers and data-nodes	Apache Spark Support Vector Machines (MLLib)	ROS (100%) + SVM-BD ROS (130%) + SVM-BD

**Table 3:** *S. cerevisiae*-*K. lactis* datasets.

Datasets	#Ex.	#Atts.	Class (maj; min)	#Class (maj; min)	%Class (maj; min)	IR
Blosum50	22.649.328	6	(0; 1)	(22.646.914; 2414)	(99.989; 0.011)	9381.489

Blosum621	22.649.328	6	(0; 1)	(22.646.914; 2414)	(99.989; 0.011)	9381.489

Blosum622	22.649.328	6	(0; 1)	(22.646.914; 2414)	(99.989; 0.011)	9381.489

Pam250	22.649.328	6	(0; 1)	(22.646.914; 2414)	(99.989; 0.011)	9381.489

**Table 4:** *S. cerevisiae*-*C. glabrata* datasets.

Datasets	#Ex.	#Atts.	Class (maj; min)	#Class (maj; min)	%Class (maj; min)	IR
Blosum50	29.887.416	6	(0; 1)	(29.884.575, 2841)	(99.99; 0.01)	10519.034

Blosum621	29.887.416	6	(0; 1)	(29.884.575, 2841)	(99.99; 0.01)	10519.034

Blosum622	29.887.416	6	(0; 1)	(29.884.575, 2841)	(99.99; 0.01)	10519.034

Pam250	29.887.416	6	(0; 1)	(29.884.575, 2841)	(99.99; 0.01)	10519.034

**Table 5:** *S. cerevisiae*-*S. pombe* datasets.

Datasets	#Ex.	#Atts.	Class (maj; min)	#Class (maj; min)	%Class (maj; min)	IR
Blosum50	8.095.907	6	(0; 1)	(8.090.950; 4.957)	(99.939; 0.061)	1632.227

Blosum621	8.095.907	6	(0; 1)	(8.090.950; 4.957)	(99.939; 0.061)	1632.227

Blosum622	8.095.907	6	(0; 1)	(8.090.950; 4.957)	(99.939; 0.061)	1632.227

Pam250	8.095.907	6	(0; 1)	(8.090.950; 4.957)	(99.939; 0.061)	1632.227

**Table 6:** Combination of alignment parameter settings on the datasets.

Dataset	Substitution matrix	Gap open	Gap extended
Blosum50	Blosum50	15	8
Blosum621	Blosum62	8	7
Blosum622	Blosum62	12	6
Pam250	Pam250	10	8

**Table 7:** Supervised algorithms and parameter values in the experiments.

Algorithm	Parameter values
RF-BD^1^	Number of trees: 100 Random selected attributes per node: 3^2^ Number of maps: 20

RF-BDCS	Number of trees: 100 Random selected attributes per node: 3 Number of maps: 20 *C*(+|−) = IR *C*(−|+) = 1

ROS (100%) + RF-BD	RS^3^ = 100%

ROS (130%) + RF-BD	RS = 130%

SVM-BD	Regulation parameter: 1.0, 0.5, and 0.0 Number of iterations: 100 (by default) StepSize: 1.0 (by default) miniBatchFraction: 1.0 (percent of the dataset evaluated in each iteration 100%)

ROS (100%) + SVM-BD	RS = 100%

ROS (130%) + SVM-BD	RS = 130%

^1^BD: big data.

^2^int⁡(log_2_⁡*N* + 1), where *N* is the number of attributes of the dataset.

^3^RS: resampling size.

**Table 8:** Unsupervised algorithms and parameter values in the experiments.

Algorithm	Parameter values	Implementation
RBH	Soft filter and Smith Waterman alignment *E*-value = 1*e* − 06	BLASTp program^1^ Matlab script

RSD	*E*-value thresholds: 1*e* − 05, 1*e* − 10, and 1*e* − 20 Divergence thresholds *α*: 0.8, 0.5, and 0.2.	BLASTp program^1^ Python script^2^

OMA	Default parameter values	OMA stand-alone^3^

^1^Available in http://www.ncbi.nlm.nih.gov/BLAST/.

^2^Available in https://pypi.python.org/pypi/reciprocal_smallest_distance/1.1.4/.

^3^Available in http://omabrowser.org/standalone/OMA.0.99z.3.tgz.

**Table 9:** Geometric mean results of the best supervised classifiers in each dataset.

Dataset	ROS (RS: 100%) + RF-BD (Scer-Klac)	ROS (RS: 130%) + RF-BD (Scer-Klac)	RF-BDCS (Scer-Klac)	ROS (RS: 100%) + RF-BD (Scer-Cgla)	ROS (RS: 130%) + RF-BD (Scer-Cgla)	RF-BDCS (Scer-Cgla)	ROS (RS: 100%) + SVM-BD (regParam: 1.0) (Scer-Spombe)	ROS (RS: 100%) + SVM-BD (regParam: 0.5) (Scer-Spombe)
Blosum50	0.9818	0.9818	**0.9896**	0.9889	0.9885	**0.9934**	0.8393	**0.8673**
Blosum621	0.9801	0.9818	**0.9855**	0.9891	0.9903	**0.9932**	0.8707	**0.8959**
Blosum622	0.9793	0.9793	**0.9905**	0.9910	0.9910	**0.9929**	0.8536	**0.8694**
Pam250	0.9818	0.9818	**0.9899**	0.9912	0.9905	**0.9941**	0.8495	**0.8839**

**Table 10:** AUC and *G*-Mean results of supervised classifiers in Experiments 1 and 2.

Algorithm	*S. cerevisiae*-*K. lactis*		*S. cerevisiae*-*C. glabrata*		*S. cerevisiae*-*S. pombe*	
	AUC	*G*-Mean	AUC	*G*-Mean	AUC	*G*-Mean
RF-BD	0.6979	0.6291	0.7455	0.7005	0.5172	0.1851
ROS (RS: 100%) + RF-BD	0.9809	0.9807	0.9901	0.9900	0.6096	0.4527
ROS (RS: 130%) + RF-BD	0.9813	0.9812	0.9901	0.9901	0.6121	0.4581
RF-BDCS	**0.9889**	**0.9889**	**0.9934**	**0.9934**	0.7294	0.6745
ROS (RS: 100%) + SVM-BD (regParam: 1.0)	0.9477	0.9477	0.9542	0.9542	0.8632	0.8533
ROS (RS: 100%) + SVM-BD (regParam: 0.5)	0.8845	0.8791	0.9540	0.9539	**0.8845**	**0.8791**
ROS (RS: 100%) + SVM-BD (regParam: 0.0)	0.6135	0.4961	0.9432	0.9431	0.6135	0.4961
ROS (RS: 130%) + SVM-BD (regParam: 1.0)	0.8164	0.7956	0.9523	0.9522	0.8164	0.7956
ROS (RS: 130%) + SVM-BD (regParam: 0.5)	0.8629	0.8528	0.9539	0.9539	0.8629	0.8528
ROS (RS: 130%) + SVM-BD (regParam: 0.0)	0.6248	0.5147	0.9429	0.9428	0.6248	0.5147

**Table 11:** Run time results in seconds of the big data solutions in Experiments 1 and 2.

Datasets	*S. cerevisiae*-*K. lactis*	*S. cerevisiae*-*C. glabrata*	*S. cerevisiae*-*S. pombe*
RF-BD	1201.59	2174.90	2060.99
ROS (RS: 100%) + RF-BD	2983.75	4562.38	4440.03
ROS (RS: 130%) + RF-BD	3345.04	4805.50	4681.51
RF-BDCS	1302.41	2362.04	2025.15
SVM-BD	**461.87**	**482.85**	**480.45**
ROS (RS: 100%) + SVM-BD (regParam: 1.0)	**867.38**	**1011.59**	**1012.46**
ROS (RS: 100%) + SVM-BD (regParam: 0.5)	**874.62**	**1008.77**	**1013.32**
ROS (RS: 100%) + SVM-BD (regParam: 0.0)	**859.17**	**1008.24**	**999.31**
ROS (RS: 130%) + SVM-BD (regParam: 1.0)	927.14	1079.19	1079.58
ROS (RS: 130%) + SVM-BD (regParam: 0.5)	929.17	1084.19	1076.33
ROS (RS: 130%) + SVM-BD (regParam: 0.0)	924.42	1076.37	1077.21

**Table 12:** AUC and *G*-Mean results of the unsupervised and the best supervised classifiers in Experiments 1 and 2.

Algorithm	*S. cerevisiae*-*K. lactis*		*S. cerevisiae*-*C. glabrata*		*S. cerevisiae*-*S. pombe*	
	AUC	*G*-Mean	AUC	*G*-Mean	AUC	*G*-Mean
RBH	0.1497	0.0062	0.8196	0.7995	0.4697	0.4525
RSD 0.2 1*e* − 20	0.5862	0.4862	0.9238	0.9206	0.4874	0.4438
RSD 0.5 1*e* − 10	0.5926	0.4643	0.9340	0.9316	0.4980	0.4063
RSD 0.8 1*e* − 05	0.5886	0.4518	0.9382	0.9362	0.5009	0.3899
OMA	0.5765	0.4904	0.9287	0.9259	0.5151	0.4644
RF-BDCS	**0.9889**	**0.9889**	**0.9934**	**0.9934**	0.7294	0.6745
ROS (RS: 100%) + SVM-BD (regParam: 1.0)	0.9477	0.9477	0.9542	0.9542	0.8632	0.8533
ROS (RS: 100%) + SVM-BD (regParam: 0.5)	0.8845	0.8791	0.9540	0.9539	**0.8845**	**0.8791**
